# The Global Health Service Partnership: An Academic–Clinical Partnership to Build Nursing and Medical Capacity in Africa

**DOI:** 10.3389/fpubh.2017.00174

**Published:** 2017-07-24

**Authors:** Eileen M. Stuart-Shor, Elizabeth Cunningham, Laura Foradori, Elizabeth Hutchinson, Martha Makwero, Jill Smith, Jane Kasozi, Esther M. Johnston, Aliasgar Khaki, Elisa Vandervort, Fabiola Moshi, Vanessa B. Kerry

**Affiliations:** ^1^Seed Global Health, Boston, MA, United States; ^2^Department of Anesthesia and Critical Care, Beth Israel Deaconess Medical Center, Boston, MA, United States; ^3^College of Nursing and Health Sciences, University of Massachusetts Boston, Boston, MA, United States; ^4^Peace Corps, Washington, DC, United States; ^5^Department of Family Medicine, Swedish Family Medicine-First Hill, University of Washington, Seattle, WA, United States; ^6^Department of Family Medicine, University of Malawi College of Medicine, Blantyre, Malawi; ^7^School of Nursing, Mbarara University of Science and Technology, Mbarara, Uganda; ^8^Wright Center National Family Medicine Residency at HealthPoint, Auburn, WA, United States; ^9^Department of Medicine, Herbert Kairuki Memorial University, College of Medicine, Dar es Salaam, Tanzania; ^10^School of Nursing, University of Utah, Salt Lake City, UT, United States; ^11^Grounds for Health, Williston, VT, United States; ^12^School of Nursing, University of Dodoma, Dodoma, Tanzania; ^13^Mass General Global Health, Massachusetts General Hospital, Boston, MA, United States; ^14^Department of Medicine, Massachusetts General Hospital, Boston, MA, United States; ^15^Department of Global Health and Social Medicine, Harvard Medical School, Boston, MA, United States

**Keywords:** global health, health professionals education, health promotion, clinical education, mentoring

## Abstract

The World Health Organization estimates a global deficit of about 12.9 million skilled health professionals (midwives, nurses, and physicians) by 2035. These shortages limit the ability of countries, particularly resource-constrained countries, to deliver basic health care, to respond to emerging and more complex needs, and to teach, graduate, and retain their future health professionals—a vicious cycle that is perpetuated and has profound implications for health security. The Global Health Service Partnership (GHSP) is a unique collaboration between the Peace Corps, President’s Emergency Plan for AIDS Relief, Seed and host-country institutions, which aims to strengthen the breadth and quality of medical and nursing education and care delivery in places with dire shortages of health professionals. Nurse and physician educators are seconded to host institutions to serve as visiting faculty alongside their local colleagues. They serve for 1 year with many staying longer. Educational and clinical best practices are shared, emphasis is placed on integration of theory and practice across the academic–clinical domains and the teaching and learning environment is expanded to include implementation science and dissemination of locally tailored and sustainable practice innovations. In the first 3 years (2013–2016) GHSP placed 97 nurse and physician educators in three countries (Malawi, Tanzania, and Uganda). These educators have taught 454 courses and workshops to 8,321 trainees, faculty members, and practicing health professionals across the curriculum and in myriad specialties. Mixed-methods evaluation included key stakeholder interviews with host institution faculty and students who indicate that the addition of GHSP enhanced clinical teaching (quality and breadth) resulting in improved clinical skills, confidence, and ability to connect theory to practice and critical thinking. The outputs and outcomes from four exemplars which focus on the translation of evidence to practice through implementation science are included. Findings from the first 3 years of GHSP suggest that an innovative, locally tailored and culturally appropriate multi-country academic–clinical partnership program that addresses national health priorities is feasible and generated new knowledge and best practices relevant to capacity building for nursing and medical education. This in turn has implications for improving the health of populations who suffer a disproportionate burden of global disease.

## Introduction and Background

Well-prepared and adequately resourced educators of nurses, physicians, and other health professionals are the basic building blocks of stronger health systems and quality education is a prerequisite for effective achievement of other investments in health infrastructure and care delivery. Despite these facts, the education of health professionals in resource-limited settings is chronically under-resourced. The World Health Organization (WHO) estimates a global deficit of about 12.9 million skilled health professionals (midwives, nurses, and physicians) by 2035 (1). These shortages limit the ability of countries to deliver basic health care, to respond to emerging and more complex needs, and to teach, graduate, and retain their future health professionals—a vicious cycle that is perpetuated and has profound implications for global health security (2–6).

## The Global Health Service Partnership (GHSP)

The GHSP is a unique collaboration between three US partners [Peace Corps, US President’s Emergency Plan for AIDS Relief (PEPFAR), Seed Global Health (Seed)], and host-country institutions. The primary purpose of GHSP is to strengthen the breadth and quality of medical and nursing education and care delivery in places with a high burden of disease and a dire shortage of health professionals. GHSP focuses on four key areas; (1) Deploying qualified and committed nurse and physician educators for 1 year to work as visiting faculty, alongside local faculty counterparts, to expand and enrich health professional education in a culturally appropriate, locally tailored and sustainable way; (2) working with partner academic institutions to identify and support urgently needed expertise and educational resources that improve the learning environment, with a priority on integration of academic–clinical teaching and learning and health-care specialties in greatest need; (3) aligning efforts and resources with the priorities of host governments and other institutions committed to health system strengthening; and (4) Providing financial assistance to and practical support for US educators that volunteer through GHSP to facilitate effective and fulfilling service.

Identifying host-country institutions with whom to partner is a multi-stage process. Peace Corps, guided by PEPFAR, identifies resource-constrained countries with a high burden of disease where they feel that GHSP can have an impact on improving accessibility to and quality of care. The Ministry of Health and/or Ministry of Higher Education in the countries of interest make the determination of whether they feel GHSP can add value to meeting their country’s priority human resources for health needs and they identify the nursing and medical training institutions where they would like GHSP to partner. Last, GHSP leadership meets with key stakeholders at the training institutions to identify their priorities and areas where they feel that seconding visiting faculty to their institution can help them meet their institutional goals. If the identified training institution wants to partner with GHSP, Peace Corps, and Seed collaborate to recruit, orient, and deploy nurse and physician educators to serve as visiting faculty at their school. GHSP educators are drawn from the US and have experience with both classroom and clinical teaching. They commit to serving for one academic year but many extend for longer periods. The GHSP program commitment to the partner institution is a multi-year commitment with structured hand-off between GHSP educators year to year. Within the framework of this partnership, the Peace Corps and Seed work closely together to provide the infrastructure, logistics and clinical/pedagogical expertise necessary for GHSP to support the deployed educators and to meet the needs of the host institution.

In the first 3 years of the program (2013–2016), GHSP placed 97 nurse and physician educators in the field to serve as Peace Corps volunteers and work as visiting faculty at the invitation of 15 academic institutions in Malawi, Tanzania, and Uganda. These educators represented 18 core medical and nursing specialties and ranged from early to late career. Recognizing that many US health professionals face financial barriers to volunteer service, Seed offers up to $30,000 per volunteer in needs-based assistance for each year served. From 2013 to 2016, more than $2.1 million dollars in debt assistance was offered as a catalyst for GHSP volunteers to serve in some of the world’s most challenging and most needed settings.

The 97 GHSP nurses and physician educators deployed in the first three years provided over 128,300 h of service on site, including: classroom and clinical teaching, skill lab pedagogy, student and faculty mentoring, continuing professional development, practice improvement projects, curricula improvements, resource and infrastructure development, community health outreach, and clinical protocol development. GHSP educators taught 454 courses and workshops to 8,321 trainees, faculty members and practicing health professionals across the curriculum (community health nursing, critical care nursing, family medicine, internal medicine, mental health, medical/surgical nursing, midwifery, obstetrics and gynecology, pediatrics, and public health). An ongoing mixed-methods evaluation of the program including interviews with host institution students and faculty suggests that GHSP educators provided enhanced clinical supervision (quality and breadth) resulting in improved student clinical skills, self-confidence and ability to connect theory to practice. Faculty reported enhancements in curricula pedagogy resulting in improved student critical thinking skills and the promotion of an open, collegial working environment.

As noted above, GHSP nurse and physician educators primarily teach in the classroom and clinical setting with an intentional emphasis on the integration of theory and practice across the academic–clinical domains. Student success is supported through a variety of pedagogical best practices that focus on translation of evidence to practice, critical thinking, and empowerment. In addition to these traditional teaching roles and strategies GHSP faculty partner with local faculty and hospital/community clinical staff to identify local practice or knowledge gaps and to propose evidence-based, locally tailored and sustainable practice improvement projects. GHSP does not identify the focus of the improvement; this is determined by the host institution(s) based on a local problem. The trigger or rationale for the improvement might be a sentinel event, high morbidity and mortality in a specific population, poor quality care or a knowledge gap. Students participate in the improvement initiatives thus integrating a culture of evidence-based practice in the next generation of African clinicians. In addition GHSP and Seed provides resource support to these academic/clinical partnerships to develop, implement, and evaluate these practice improvement projects as well as to host-country conferences which value and highlight local science and best practices. These service-oriented activities extend beyond the classroom to provide opportunities for academic faculty and practicing clinicians to work together to have a direct and immediate impact on the health of the population. The projects also create a fluid teaching and learning environment and contribute to enhanced opportunities for student learning.

The following four exemplars demonstrate efforts that emanate from a national health priority, that have contributed to the development of clinical and academic excellence, and translate evidence to practice through implementation science. The projects highlighted in this manuscript were intended to improve care at the point of service and did not test new theories or procedures. Therefore the projects do not meet the criteria for human subjects research and formal ethics review was not sought.

## Exemplars

### The Family Medicine Training Program in Malawi

#### Local Problem

Malawi, similar to other resource-constrained countries in Africa bears a disproportionate burden of disease in the context of threats from new and emerging infectious diseases and an epidemiologic shift toward the high prevalence of non-communicable disease (7–9). To address these needs, the WHO has advocated for a strong primary care health system at the district level that cares for patients across the lifespan (10, 11). Family medicine, a core contributor to primary health care, is critical to the achievement of equitable health outcomes for all (11) and this is a pivotal time for family medicine expansion globally. Many countries are reorienting family medicine as the cornerstone of their health-care system and expanding family medicine training. Although the practice of family medicine will have distinct characteristics around the world, the core values remain: providing care that meets the fundamental health needs of the patient, is integrated, is prioritized, and offers healing above cure when appropriate.

#### What We Did

Countries introducing family medicine, such as Malawi, face many challenges in integrating this broad discipline into their specialty-based medical system. Starting in 2014, two Malawian family medicine faculty at the University of Malawi College of Medicine (CoM) launched their first class of family medicine residents at a new district hospital training site. The goal of this program was to meet the national need for an enhanced primary care system. Not only did they create new curriculum, certify the program with the Malawi Medical Council, determine training sites, and recruit clinical faculty, but they also worked to create a new medical culture. In collaboration with the CoM Department of Family Medicine in Malawi, GHSP and Seed sent family medicine physician educators to support this new academic endeavor.

In addition to faculty support, family medicine residents engaged with a range of learners and teachers, which fostered teamwork and promoted exchange of knowledge and ideas. This includes US residents, who worked with GHSP faculty to augment their efforts to build an academic learning environment at the district training site. Senior family medicine residents from University of Washington-affiliated programs committed to working in Malawi for 9 months of the year for 5 years. US residents travel two at a time to Malawi for 4-week rotations. Their work is to participate in clinical care alongside the Malawian residents and students (from several health science disciplines), conduct bedside and didactic teaching, and support quality improvement and research projects. After the US residents receive an extensive pre-travel orientation, they work under the supervision of both GHSP and in-country faculty. There is opportunity for ongoing monitoring, evaluation, and frank discussions about mutual benefit for both the host country and the visitors.

#### What We Found and What It Means

The program was evaluated after the first year using anonymous surveys given to Malawian trainees, staff and faculty. Six questions were asked, half of which were Likert scale and the other half free response. On a scale of one to five, the reported impact on medical knowledge, clinical skills, medical student teaching, and medical student supervision averaged 4.3/5. Regarding registrar and faculty mentorship and training, scores were 3.64/5 and 3.5/5, respectively. Regarding improvement of clinical care on the wards, the visiting residents were given a 4.68/5. Comments included: “*Patients’ care was directly improved.” “Misdiagnoses were corrected.” “Better treatment plans were made.” “Seeing patients today created opportunities for in-depth teaching.”*

Regarding impact on morale and the overall learning environment in the hospital, responses averaged 4.37/5 and 4.44/5, respectively. There was a slight positive response to assistance with research projects in the hospital and improved understanding of the principles of family medicine. Mostly positive comments about the partnership were made illustrated by the excerpts noted below:

They [US residents] put a very positive impact on the learning environment because each time you approached them with your problem, they were eager to help you and would exhaust every knowledge that they have that made the learning environment favorable.

They have improved my zeal for clinical medicine; They reminded me to improve my clinical skills based on the discussions we had with several patients.

They are excellent teachers who took their time to make sure the students completely understood the lesson being taught.

They helped me understand clearly about family medicine, which I did not quite understand it.

Boosted morale knowing you can ask for help any time.

They gave us inspiration that we can treat our patients using the locally available resources.

Malawi’s efforts to put broadly trained family medicine doctors in the critically important district hospital setting where many patients first seek care is based on more than 30 years of strong evidence. This strategic process and thoughtful approach is progressive and has the potential to set a precedent for other nations in the region. Malawi has the vision and desire to bring family medicine to the rural parts of its country but in this resource-scarce setting, the startup capital (human and material) can be prohibitive. The GHSP Family Medicine initiative provides reciprocity and bidirectional benefits to accrue to all of the partners (GHSP, the host institution and US Family Medicine Program). Specifically the initiative adds value to the clinical and educational mission of the host-country training program without overburdening local faculty while also providing a meaningful high quality global health experience for US Family Medicine residents and allows GHSP to fulfill its core vision of strengthening medical education and care delivery in a resource-constrained setting.

### “Redressing the Wound” in Uganda: A Deeper Look at Burn and Wound Care

#### Local Problem

When the Uganda GHSP nurse educators began accompanying nursing students on their clinical rounds at Mbarara Regional Referral Hospital (MRRH) they were distressed by the lack of priority given to treating/controlling pain particularly during dressing changes. This was especially baffling as Mbarara is one of the three primary centers where Hospice Africa-Uganda works and this would seem to indicate an awareness of the importance of palliation of pain in this region. To understand this paradox GHSP visiting faculty in collaboration with students reached out to patients, local faculty and hospital staff, and through these discussions several important barriers to effective pain management emerged including staffing and supply constraints, fear of addiction, and a lack of knowledge regarding pain management (e.g., a belief that alleviation of pain causes wounds to heal more slowly). In the surgical ward, GHSP educators and their students also witnessed poor wound-management practices and a severe shortage of wound-care supplies that prevented appropriate and timely dressing changes. These observations are consistent with reports in the literature that in resource-constrained settings, simple pain regimens, typically relying on inexpensive drugs, are often not followed due to inadequate health-care systems (12). Specifically it has been noted that pain management appears to have a lower priority than other aspects of health care in resource-constrained settings due in part to a lack of medications but also to misunderstandings about drug side effects and the fact that staff have become so accustomed to severely limited resources to treat patients in pain that non-treatment becomes the norm. Patients themselves often believe that nothing can be done and may suffer in silence (12, 13). Similar constraints in the treatment of burns in resource-limited settings, particularly in the pediatric population, have been noted in the literature (14, 15). Students, faculty, hospital staff, and GHSP educators agreed; it was time to do something.

#### What We Did

To that end, GHSP educators and their host-country counterparts across multiple Ugandan training institutions organized an interdisciplinary conference bringing together nurses, physicians and students from all GHSP-partner institutions to discuss locally relevant best practices in pain management, wound and burn care. Emphasis was placed on drawing from local expertise to review current published best practices in pain management but, more importantly, to stimulate active discussion among the participants around locally tailored solutions. The goal was to highlight local best practices and to identify knowledge, practice, and research gaps in order to chart a way forward. Inclusion of students (the future of evidence-based practice) and interdisciplinary care were also prioritized. The GHSP educators assumed responsibility for the logistical support. Several planning meetings for the conference were attempted *via* Skype, with mixed success, resulting in most of the conference planning conversations being informal and spontaneous.

#### What We Found and What It Means

The conference was held in northern Uganda with over 200 health professionals from around the country attending. Participants included hospital nurses and physicians, university lecturers, hospital and university administration, and many medical and nursing students. Engagement in the conference activities was high and post-conference evaluations were strongly positive. The group from Mbarara University of Science and Technology (MUST) and MRRH (including hospital and university personnel) were very excited to visit northern Uganda and curious to see the facilities there and meet their northern Ugandan counterparts. MUST nursing students were excited to be afforded the opportunity to attend the conference and to be treated as the budding professionals they would soon be. In addition, the Master’s level nursing students appreciated the opportunity to meet other professional nurses and network with potential employers. Overall, both nursing and medical students were engaged in the conference and actively participated in the question and answer sessions. It was powerful for the Ugandan nurses to see the keynote speaker (a Uganda physician) ask a Ugandan nurse colleague to join him at the speaker’s podium to jointly answer questions from the conference attendees.

Post-conference evaluations noted that teamwork was emphasized in efficient and effective pain management, as was the necessity of having the will to critically assess the wound to determine the method of dressing and documentation. As a way forward from the discussions, participants saw that there was a need to set up burns units or cubicles in different hospitals to maintain asepsis that will promote healing. One participant noted that “Best practice is the goal for every day performance on a wound.” Networking and interactions with different health workers from other institutions was valued as an opportunity to make a difference in redressing the wound. The following quotes emanating from the conference summarizes the intentions of wound care by individual nurses in Ugandan hospitals.

You can’t change the whole world, and you certainly can’t change other people, but you do have the ability to change yourself. You can have a positive impact in redressing the wound in a pain free environment, the communication and comfort to patients that you dress, and the entire community…the choice is up to you.

As a team, let us flash pain down the drain while re-dressing the wound.

Outcomes from this conference illustrate the value added of supporting local conferences. Networking, sharing of best practices, valuing local science and practice, cross-disciplinary discussions, and inclusion of students all led to a culture of commitment to improving pain management and wound care that will directly and immediately benefit the care of everyday Ugandans. In addition, the conference led to partnerships culminating in grant applications for both practice improvement and research in this important area. At least one research application was funded and that work is underway.

### Neonatal Resuscitation Program (NRP) in Tanzania

#### Local Problem

It has been estimated that worldwide 6.3 million children under the age of 5 years died in the year 2013 alone, and notably 44% of those deaths occurred in the first 4 weeks of life (16). In Tanzania, 2013 country-level estimates report that 21 newborns died out of every 1,000 births. The Every Newborn Action Plan, endorsed by governments, the private sector, civil society, and other stakeholders, calls for reducing neonatal mortality rates in all countries to fewer than 10 deaths per 1,000 live births by 2035 thus providing a benchmark for Tanzania to aspire to Ref. (16). UNICEF has suggested that up to 30% of newborn deaths could be prevented with access to essential care, including basic resuscitation. Despite country-specific high infant mortality rates, evidence that access to basic resuscitation could significantly decrease newborn mortality, and national recognition of the importance of this training, neonatal resuscitation has not historically been well-integrated in medical and nursing school education in Tanzania (17). In addition to lack of prioritization of the problem, supplies to conduct resuscitation properly are frequently unavailable (18).

#### What We Did

In 2014–2015, GHSP expanded to a new site in Dar es Salaam, placing educators in medicine and nursing at the Hubert Kairuki Memorial University (HKMU) School of Medicine and HKMU School of Nursing. Within the first few months on-site a student approached one of the GHSP educators to raise concerns about the frequent neonatal deaths witnessed every day in the nursery at the local regional hospital. Countless infants faced respiratory distress at birth, but local hospital staff and learners who rotated through the facility lacked knowledge and supplies to resuscitate these newborns.

One GHSP physician educator reached out to a local NGO conducting a Helping Babies Breathe master training session in Dar es Salaam to obtain a training manikin and educational materials. Starting with medical students on their fourth-year pediatrics rotation, a multi-session training course was offered in which students learned about the philosophy and principles of neonatal resuscitation following the Neonatal Resuscitation Program (NRP) (19). Students learned about teamwork and assignment of roles during resuscitation. They embraced the underlying principles for each step in the protocol and practiced the actual skills extensively using manikins. When it became clear that hands-on skills training was both desired and effective in teaching the students, additional financial support was requested and received from Seed and GHSP to purchase 11 additional manikins.

Following the initial training sessions, the HKMU Student Association recognized the need to teach and train other students across the medical and nursing school to perform neonatal resuscitation. Over three separate sessions, several dozen committed nursing and medical student leaders attended “train the trainer” workshops in which they received extensive training on both the NRP protocol and how to effectively teach resuscitation skills. Members of the fourth-year pediatrics rotation who had previously been trained served as assistant teachers for these sessions. On April 24, 2015, these committed student leaders organized and led a school-wide continuing medical education (CME) workshop on neonatal resuscitation, attended by a local academic leader and advocate for neonatal resuscitation training in Tanzania. The students offered a detailed lecture presentation and hands-on skills training to over 200 students, faculty, and staff nurses and physicians from the local private and regional public hospital. At the workshop, one local physician approached the GHSP physician educator to inform her that a baby the fourth year medical students had resuscitated the day before was alive and well, thanks to their efforts.

#### What We Found and What It Means

Since the initial CME workshop, HKMU students have gone on to lead a skills workshop for a local Medical Students Association, a training session at Mwananyamala Regional Hospital (funded by Jhpiego), and another CME workshop for the HKMU community. All told, these students have trained over 600 of their fellow students and local health-care workers in neonatal resuscitation techniques. One of these student leaders has gone on to obtain formal Master Trainer certification from Helping Babies Breathe and subsequently trained fourth year medical students at HKMU. The current student leaders at HKMU are committed to training new trainers in order to ensure sustainability of their resuscitation training. Pre- and post-workshop quizzes continue to provide evidence that knowledge level improve as a result of the training and ample anecdotal evidence from community health-care providers indicates that these trainings have translated to lives saved in the nursery as providers become more confidant and effective in implementing neonatal resuscitation techniques. One of the students was recently at a private hospital when a neonate coded; the nurse present started resuscitation, however the student noticed the resuscitation wasn’t according to protocol and stepped in to offer help; the baby survived. Soon after the nurse asked the student to help her refresh her skills and they were able to discuss the correct steps of resuscitation. The art of this training is that it has a direct effect to the neonate as well as demonstrates the power of interdisciplinary learning.

### Cervical Cancer Prevention Training in Tanzania

#### Local Problem

Cervical cancer is the most common female cancer in sub-Saharan Africa. Of all new cervical cancer cases in Africa, it is estimated that 40% of new cases occur in Eastern Africa. Tanzania has one of the highest rates of cervical cancer and it is the leading cause of cancer *and* cancer death among women ages 15– 44 (20). Worldwide, more than 85% of cervical cancer deaths are in low-resource settings, such as Tanzania. This is despite the fact that cervical cancer is preventable—with early detection and treatment of pre-cancerous lesions and early cancers (21, 22). Women die in such large numbers because most are diagnosed in advanced stages of cervical cancer, when treatments are no longer available (23, 24).

In Tanzania there has been a considerable cervical cancer prevention effort over the past 20 years (23, 25). Tanzanian health-care providers are trained in visual inspection with acetic acid (VIA) and cryotherapy per the Tanzanian Ministry of Health and Social Welfare (MOHSW) Guidelines (24, 26). This is an effective, affordable screening and treatment option for pre-cancer of the cervix and echoes the guidelines from the WHO for low-resource settings (24). Despite these efforts to scale-up cervical cancer prevention services, screening and treatment coverage has not yet been achieved and a high disease burden persists (20, 27).

To bridge the gap in coverage, Ministries of Health and the international community are developing novel, cost effective, locally applicable training, screening and treatment approaches for low-resource settings (27–31). Regardless of the technology used to screen and treat cervical pre-cancer, however, frontline health workers require specialized training to provide this life-saving service and many challenges to both training and maintaining core competencies in this area have been reported in the literature (27–31). This suggests that including this content in pre-service education might be important. At present, most pre-service nursing and midwifery students in Tanzania do not receive training in cervical cancer prevention with VIA and cryotherapy. To that end, a GHSP nurse educator and faculty at the University of Dodoma piloted a program to train Tanzanian pre-service students of nursing and midwifery in core knowledge for cervical cancer prevention using VIA and cryotherapy.

#### What We Did

The first steps taken were to conduct a needs assessment of cervical cancer prevention screening and focus on relationship building with providers and trainers both regionally and nationally. This process included a broad review of local MOHSW resources and NGO partners engaged in cervical cancer prevention efforts. Next, through funding support from Seed and GHSP and collaboration with the local HIV implementing partner, the team ensured that local clinical sites had crucial supplies required for performing VIA and cryotherapy.

The team then conducted a refresher training in VIA and cryotherapy for local MOHSW trained providers and key district and university leadership. The training evaluated skills and learning needs of clinical providers and initiated a process to strengthen foundational knowledge about VIA and cryotherapy. This 1-day didactic conference also introduced key university nursing/midwifery faculty to the cervical cancer prevention content and laid the groundwork for future training of pre-service nursing and midwifery students in cervical cancer prevention at these sites. Once the clinical MOHSW sites were engaged, refreshed and equipped, the pilot shifted focus to the training of upper year bachelors and diploma students at the University of Dodoma.

In total, 187 pre-service nursing and midwifery students completed a 1-day didactic training in cervical cancer prevention with a focus on VIA and cryotherapy. Of those students trained, 27 were diploma candidates in their final year of training. The remaining 160 were third- and fourth-year bachelor’s students. The didactic training was an interactive training adapted from high quality training materials from the Tanzanian MOHSW National Training, Jhpiego, PATH and Grounds for Health.

Currently, clinical training in VIA and cryotherapy is underway for those pre-service students who completed the didactic training by nurses trained and certified by the Tanzanian MOHSW. While they will not be certified to independently perform VIA and cryotherapy, they will have received foundational training that they will apply in their future practice, ideally in locations where cervical cancer prevention programming is already in process.

#### What We Found and What It Means

As part of the initial assessment, the team found that student nurses and midwives had little prior knowledge about cervical cancer, basic cervical anatomy including variations of normal, or how to perform speculum and bimanual exams. Yet, students were eager to acquire any possible knowledge and skills to improve the health of their communities, especially life-saving preventive services that can be provided by nurses and midwives in remote areas. Despite strong University and MOHSW interest in cervical cancer prevention, urgent and competing priorities made it difficult to prioritize the integration of this vital life-saving care into the health system, which makes it all the more important to integrate this training into the pre-service curriculum.

Challenges to scaling-up this education are myriad. In sub-Saharan Africa, cervical cancer incidence is impacted by the HIV/AIDS pandemic. As of 2014, the UN estimated that 24.7 million people are living with HIV in sub-Saharan Africa, 58% of whom are women (24). Women living with HIV have a higher prevalence and incidence of cervical pre-cancer compared to HIV-negative women (32). Thus, women living in this region are at higher risk for cervical cancer and this is occurring in the tandem with constrained and decreasing cervical cancer prevention and treatment funding (20, 27). This double burden of high prevalence and few resources requires new, clinically and cost-effective approaches to the sustainable cancer prevention training of health professionals. In that context, building cervical cancer screening education into the pre-service curriculum is an important step and helps achieve Tanzania’s goal to address cervical cancer while also increasing the awareness and clinical knowledge of the soon to be graduates (32).

To this end, further capacity building efforts focused on nursing and midwifery educators in Tanzania are crucial. These educators are well positioned to provide the pre-service didactic content but will need support from expert MOHSW trainers and care providers to assure that the information and skill-building provided to students is state-of-the art and evidence based. Regardless of the myriad challenges, access to life-saving reproductive health care for all women, especially in the global south, is a human right and must be a priority (27).

## Discussion

Findings from the first 3 years of GHSP suggest that an innovative, locally tailored and culturally appropriate multi-country academic partnership program is feasible and generated enhancements in nursing and medical education as well as the implementation of practice improvement initiatives relevant to capacity strengthening for nursing and medical education. The exemplars demonstrate that twenty-first century health professional education needs to be responsive to national and local health priorities and be creative in developing teaching and learning strategies that are linked to service and cross the academic/clinical domains. Key features of the GHSP program emphasize and support these needs, including the intentional pairing of the US/African educators, emphasis on faculty supervised clinical instruction, involvement of students in academic enrichment activities such as practice improvement projects, and a sustained commitment to host-country institutions over time. Although GHSP nursing and physician educators are seconded to academic institutions and teach primarily in the classroom and clinical setting, access to support (financial and pedagogical) for practice improvement projects contributes to their ability to create a fluid learning environment for students and staff across academic–clinical settings. Continued evaluation of the model will help inform optimizing classroom and clinical pedagogy in resource-constrained settings to both improve the health and well-being of populations who suffer a disproportionally high burden of disease, as well as to demonstrate the retention of health-care providers in a system in which they feel invested and able to make an impact.

The body of work describe in this manuscript focuses on translating evidence to practice and as such does not attempt to create new knowledge that is generalizable. Rather, our work addresses a practice gap by implementing evidence-based teaching/learning strategies and clinical practice interventions to achieve an immediate improvement in education or care within the local context. Accelerating the uptake of evidence into the education and practice environment has been identified as a priority for translational science. The GHSP outcomes and examplars described in this manuscript demonstrate that with attention to local context and culture, evidence-based best practices can be implemented in health professionals training and clinical practice even in resource-constrained settings.

An important lesson learned from the Ebola crisis is that global health security is dependent on individual health security and that individual health security is dependent on having a sufficient number of qualified health professionals and faculty (6). It is not enough to have a sufficient pool of health professionals; they must be well prepared to respond to existing and emerging health threats in their country. To do that requires that we move outside traditional pre-service/in-service silos and create dynamic learning environments that promote critical thinking, that empower students and clinicians to tailor best practices to meet local context, and that challenge students and clinicians to think across disciplines and across the care delivery domains. Challenging the norm and charting new models to address primary care, pain and wound management, neonatal mortality, and cervical cancer prevention required the will to address a current health threat, to not be constrained by the reality of scarce resources or the *status quo*, to work across the academic–clinical domains and to feel empowered to act. GHSP, with its focus on assisting academic institutions, health-care facilities, and individual faculty and students to create a culture of academic/clinical excellence, is well positioned to collaborate with these host-country partners to achieve their aspirational goals.

## Author Contributions

ES, EC, LF, and VK conceptualized the manuscript, were intimately involved in the work described in the manuscript, and worked on the drafts and final version of the manuscript. EH and MM wrote the draft for the family medicine exemplar and approved the final draft. JS and JK wrote the draft for the wound care exemplar and approved the final draft. EJ and AK wrote the NRP exemplar and approved the final draft. EV and FM wrote the cervical cancer exemplar and approved the final draft.

## Conflict of Interest Statement

The authors declare that the research was conducted in the absence of any commercial or financial relationships that could be construed as a potential conflict of interest.
